# Sex-Related Differences in Plasma Oxytocin Levels in Humans

**DOI:** 10.2174/1745017901915010058

**Published:** 2019-03-26

**Authors:** Donatella Marazziti, Stefano Baroni, Federico Mucci, Armando Piccinni, Ilenia Moroni, Gino Giannaccini, Claudia Carmassi, Enrico Massimetti, Liliana Dell’Osso

**Affiliations:** 1 Dipartimento di Medicina Clinica e Sperimentale, Section of Psychiatry, University of Pisa, Pisa, Italy; 2 Dipartimento di Farmacia, University of Pisa, Pisa, Italy; 3 ASST, Bergamo Ovest, SSD Servizio Psichiatrico diagnosi e cura, Treviglio, Italy

**Keywords:** Dymorphic changes, Humans, Oxytocin, Plasma levels, Social emotions, AVP receptors

## Abstract

**Background:**

Increasing evidence supports a key role of Oxytocin (OT) as a modulator of social relationships in mammals.

**Objective:**

The aim of the present study was to investigate possible sex-related differences in plasma OT levels in human beings.

**Methods:**

Forty-five healthy men and 45 women (mean age: 34.9 ± 6.2 years), were included in the study. Plasma preparation, peptide extraction and OT radioimmunoassay were carried out according to standardized methods.

**Results:**

The results showed that OT plasma levels (pg / ml, mean ± SD) were significantly higher in women than in men (4.53 ± 1.18 vs 1.53 ± 1.19, p ˂ 0.001).

**Conclusions:**

The present finding demonstrates sex-related differences in plasma OT levels in humans. It is tempting to hypothesize that such differences might be related to behaviours, attitudes, as well as susceptibility to stress response, resilience and social emotions specific of women and men.

## 1 INTRODUCTION

Oxytocin (OT) is produced in the magnocellular neurons of the paraventricular and supraoptic nuclei of the hypothalamus and is released in the blood stream by the posterior pituitary gland [1]. Although related to other peptides present in all vertebrates, OT appears to be an exclusive mammalian hormone, and it plays a key role in labor and lactation, as well as in the onset and in the maintenance of a wide range of social behaviours [2]. The evidence of an intimate connection between OT and the development of complex social behaviours is rising: as a matter of fact, OT has been reported to promote social approach, pair bonding and copulation throughout a modulation of neuroendocrine and behavioural responses to social stress [3]. Not surprisingly, currently OT is supposed to be one of the main mediators of the development of attachment in childhood, pair bonding, as well as to relating social signals with cognition, behaviours and reward [4-7]. Besides the animal data, converging findings are accumulating highlighting the increasing role of OT as a prosocial mediator in human beings [6-8]. Some authors found out that early social experiences in humans are capable of influencing the OT system but also the Arginine-vasopressin (AVP, another hypothalamic protein hormone that might be exerting opposite functions to OT) system [9, 10]. In particular, the available evidence suggests that proper development of the OT and AVP systems may be critically impaired by a lack of the essential care in young children. Furthermore, a dysfunctional functioning of those systems seems to result in a disruption of the typical calming and comforting effects provided from the caregivers [11, 12]. Not surprisingly, comforting physical contact, familiar smells and other socially pleasant sensory experiences are capable of raising the levels of OT and AVP [4, 11, 12].

Interestingly, the OT system and some of its parameters show some sex-related differences depending on the brain region and species. In rats, the density of OT-immunoreactive axons tends to be higher in females [13]. It was also observed that OT Receptor (OTR) binding seems to be increased up to 40% by a high maternal licking and grooming in females, while AVP receptors of type 1a (APVRs) are more evident in males [14]. The results of different studies carried out on several animal species, which show more prominent effects of OT in females and more significant effects of AVP in males, appear congruent with the dimorphic effects on OTR and V1a receptors [15, 16]. Similarly, pair bonding in monogamous praire voles seems apparently to depend on OT in females and on AVP in males, at variance with polygamous montane voles [13, 16-19]. The different behaviours are possibly the result of the interaction of OT and AVP with steroid hormones, such as estradiol and progesterone: in particular estradiol enhances OTR affinity, while progesterone decreases it, especially in the amygdala [20-22].

Data in humans are more limited and mainly indirect. The administration of intranasal OT enhanced the recognition of kinship in women and of competition in men [23]. Moreover, this procedure seems to reduce amygdala activation in men and to increase it in women when observing emotional facial expressions [24]. Generally, women consider all human faces more positive and appealing than men, while showing a preference for facial beauty of children and elderly people [25]. Gender differences in the neural and behavioural response to OT administration during human social interaction have been also reported [26]. In men performing cooperative interactions, OT and AVP were shown to enhance brain areas abundant in OTRs and AVPRs and/or related to reward, social bonding, arousal and memory. By contrast, in women, OT and AVP showed decreased activity or no effect in these areas. Somehow, OT treatment turned neural responses of men more similar to those of women in the placebo group and *vice-versa* [27]. The increase of OT levels while facing interpersonal distress or psychological stress is also more marked in women and possibly mediated by sex hormones, as estrogens are supposed to up-regulate the OT system with a resulting enhancement of social interest and approach behaviours [22, 27]. For this reason, they are considered an index of stressful relationships in women, but not in men, where AVP would play a major role [28]. Indirect support derives also from the clinical evidence of a female prevalence of both major depression and social anxiety disorder, highly associated with social–emotional stress [3, 29, 30].

Taken together, we may reasonably speculate that the OT system could act distinctly in men and women through the possible mediations of sex hormones [31-33].

The limited literature on the topic of dymorphism of OT system in humans is with no doubt related to the lack of reliable peripheral parameters reflecting its central functioning. Plasma OT levels are considered a reliable marker of the CNS concentrations of this neurohormone [4, 27, 34, 35], although disagreements do exist [16]. However, a series of reports on parallel changes in OT levels in plasma and in the brain [4, 27, 34] would suggest common regulatory processes and, thus, support the use of the peripheral OT as a mirror of the central one [35].

Therefore, due to the limited available data, this study aimed at measuring and comparing plasma OT levels in a sample of healthy men and women.

## 2 MATERIALS AND METHODS

Forty-five men (mean age: 35.1 ± 7.5 years) and 45 women (mean age: 34.9 ± 6.2 years), recruited amongst medical and nursing staff at the Dipartimento di Medicina Clinica e Sperimentale, Section of Psychiatry, University of Pisa. All subjects who volunteered for the study underwent a detailed medical interview, conducted by senior psychiatrists (DM and AP). None of the subjects had any personal or family history of major psychiatric disorders or medical diseases. No subjects reported any regular intake of psychotropic or other medical compounds. Finally, none of them reported to be a heavy cigarette smoker or alcohol drinker. All subjects were within the normal body/mass index (BMI) range. All women had a normal menstrual cycle and did not take any contraceptive pills; all their blood samples were collected in the early follicular phase (between the second and the fifth day of the menses). No past history or current signs/symptoms of hypogonadism or disease of genital system was detected in men. Thirty-nine men and 30 women were involved in a love relationship, of whom ten and twelve, respectively, were married, while two were separated/divorced, and the remaining were single. All subjects had completed the high school, 25 men and 23 women were graduated and/or PhD. All the information derived from the medical history collected by one of the authors (DM).

All subjects provided the written informed consent to participate in the study previously approved by the Ethics Committee at Pisa University.

### 2.1 Plasma Preparation

In the periods January-March, and between 8 and 10 am, 10 ml of blood was withdrawn three times from an antecubital vein from fasting subjects who were sitting and relaxing in the same setting at a constant temperature of about 19° C. The blood (10 ml) was collected in vacutainers with EDTA as anti-coagulant, transferred to centrifuge tubes containing aprotinin (Sigma, Milan, Italy) (0.6 TIU/ml of blood), and gently mixed several times to inhibit the activity of proteinases. Blood was then centrifuged at 1,600 x g for 15 min at 4°C and the ensuing plasma was collected and kept at -70°C until the assay.

### 2.2 Extraction of Peptides from Plasma

6 ml of plasma was acidified with 6 ml of buffer A (1% trifluoroacetic acid in H_2_O), centrifuged at 17,000 × g for 20 min at 4°C and the ensuing supernatant was collected. C-18 sep-columns were equilibrated with 1 ml of buffer B (60% acetonitrile in 1% trifluoroacetic acid) followed by buffer A (3 ml, 3 times). Acidified plasma solution was loaded into the pre-treated C-18 Sep-column washed with buffer A (3 ml, twice). Oxytocin was then eluted with buffer B (3 ml, once) and collected into a polystyrene tube. The eluate was evaporated in a Speedvac centrifugal concentrator, and the remaining product was lyophilized by a freeze dryer.

### 2.3 Oxytocin Radioimmunoassay

Radioimmunoassay (RIA) was performed by a Phoenix Pharmaceuticals Oxytocin RIA kit (Belmont, California, USA) according to our method [36]. The assay sensitivity, measured as IC50, was 10-30 pg/tube, and intra-assay and inter-assay values were 9% and 11%, respectively. Lyophilized samples and standard OT were rehydrated with RIA buffer, and dilutions of standard oxytocin were prepared (from 1 to 128 pg/tube). Primary rabbit anti-OT antibodies were added to both sample and standard, and subsequently, the mixtures were stored for 24 hours at 4°C. ^125^I-Oxytocin was then added to mixtures that were stored for 24 hrs at 4°C. Goat anti-rabbit IgG serum and normal rabbit serum were added to each tube. Afterwards, tubes were centrifuged at 1700 × g for 20min at 4°C. All the supernatant was carefully aspirated and the pellets were counted by a gamma-counter (Wizard, Perkin Elmer, Milan, Italy). All samples were assayed in duplicate. Standard curve and calculations of unknown samples were performed by means of Graphpad Prism3 software.

### 2.4 Statistical Analyses

The Student t-test (two-tailed unpaired) was used to assess the intergroup difference of age and OT levels, by means of the SPSS, Version 12.0.1 (SPSS Inc., 2003).

## 3 RESULTS

The plasma OT levels (pg/ml, mean ± SD) were significantly (p < 0.001) higher in women (range between 2.34 ± 0.02 and 6.59 ± 0.01, mean ± SD: 4.53 ± 1.18) than in men (range between 0.14 ± 0.02 and 2.59 ± 0.01, mean ± SD: 1.53 ± 1.19) each single value represented the mean of three evaluations performed within one hour (Table **1**).

Plasma OT levels were not different in married or single subjects, while, for the small sample size, no comparisons could be performed in individuals with and without a partner.

## 4 DISCUSSION

Given the paucity of information, this study aimed at investigating the possible existence of sex-related differences in OT plasma levels in humans. The ensuing findings indicate that women showed higher OT levels than men. To our knowledge, this is one of the first demonstrations of a clear-cut difference in peripheral OT between the two sexes in humans. Previously, in most cases, no sex differences were found [36-39], but there is also evidence of elevated levels in men [40]. Only one study [41] reported higher OT levels in older women than in men.

Our data, with the limits of a peripheral, albeit reliable parameter, can be considered in agreement with those mainly gathered in animals, while supporting the notion that behavioural differences in social and relationship contexts between the two sexes might be related to OT concentrations, functioning or processing [13, 18, 19, 42-44]. Another limitation of our study was that estradiol levels were not evaluated while being a potentially confounding variable. Again, the sample size was small, with all subjects belonging to the same environment, with similar educational level and lifestyle: these characteristics may limit the generalizability of our findings.

Until now, the available evidence in humans has been mainly indirect and based on a few observations of behavioural and emotional differences between men and women of in kinship, competition skills or facial recognition following intranasal OT challenges [23-25]. In addition, it should be noted that generally previous studies are affected by a sex bias, as a large majority of them included only men as participants [26].

It is conceivable that OT might have refined gender-specific functional roles within the context of human sociality: it is noteworthy that intranasal OT administration has been shown to increase amygdala activation in men facing aggressive individuals, while in women it occurs when they observe individuals approving somebody else. Therefore, according to this result, it was concluded that OT plays a specific role in promoting the positive and negative salience of a social encounter, respectively, in women and in men [45].

Reported sex differences have been attributed to the interactive effect of OT with sex steroid hormones, particularly estrogens [46]. Previous studies demonstrated that neuro-endocrine activation depends on the stressor and on many other factors including the individual's sex [47]. Some authors claimed that, since OTRs exhibit a diverse expression within the rodent brain, the receptors might play a complementary or opposing role in the gender-specific regulation of social behaviour [48].

Indeed, a properly regulated OT system has been suggested to enhance resilience and to act as a hurdle against the development of mental disorders and/or substance abuse [49, 50]. Accumulating evidence suggests a role for OT in the pathophysiology of different psychopathological conditions, mainly in those characterized by social and emotional deficits [3, 29, 30, 51, 52], such as depression [53-56], social phobia [57], anxiety disorders [3], autism spectrum disorders [58, 59], OCD [60, 61], post traumatic stress disorders [9], and other conditions [61-63] almost all characterized by a female preponderance [64, 65]. Again, sex-related OT differences have been related to emotional distress [28, 66] and to the anxiety of a love relationship [36, 67]. Recently, a common OTR gene (rs53576) polymorphism has been associated with empathic concern and emotional aspects of empathy, with women showing higher empathy scores than men [68].

## CONCLUSION

It is tempting to speculate that OT might be considered as one of the main modulators of some specific behaviours and attitudes (stress response, social skills, altruism, trust, empathy, love) that may be different in the two sexes [31, 69-71]. Interestingly, salivary OT has been positively related to generosity in young girls [72].

In any case, although our sample size was small and quite homogenous, the present findings would indicate that women show higher plasma OT concentrations than men. Further studies are needed to replicate or not this finding in larger samples composed by more heterogeneous subjects, as well as to evaluate if differences in OT concentrations represent an indicator of a different susceptibility to stress response, resilience and social emotions that are, however, functional for creating and sustaining an optimal biosocial environment.

## Figures and Tables

**Table 1:** Demographic data and plasma oxytocin (OT) levels in healthy men (M) and women (W).

	Age (years)	Plasma OT (pg/mL, mean ± SD)
Total HC (90)	35.0 ± 6.4	2.24 ± 1.99
M (45)	35.1 ± 7.5	1.53 ± 1.18*
F (45)	34.9 ± 6.2	4.53 ± 1.18

Age (years). OT (pg/ml). * significant: p < 0.001.

## LIST OF ABBREVIATIONS

AVP: = Arginine-vasopressin
AVPR(s): = AVP Receptor(s)
BMI: = Body/Mass Index
EDTA: = Ethylenediaminetetraacetic Acid
OT: = Oxytocin
OTR (s): = OT Receptor (s)
RIA: = Radio-immuno Assay
